# Natural Killer Cells in Kidney Health and Disease

**DOI:** 10.3389/fimmu.2019.00587

**Published:** 2019-03-26

**Authors:** Jan-Eric Turner, Constantin Rickassel, Helen Healy, Andrew J. Kassianos

**Affiliations:** ^1^III Department of Medicine, University Medical Center Hamburg-Eppendorf, Hamburg, Germany; ^2^Conjoint Kidney Research Laboratory, Chemical Pathology–Pathology Queensland, Brisbane, QLD, Australia; ^3^Kidney Health Service, Royal Brisbane and Women's Hospital, Brisbane, QLD, Australia

**Keywords:** natural killer cells, acute kidney injury, glomerulonephritis, chronic kidney disease, transplantation

## Abstract

Natural killer (NK) cells are a specialized population of innate lymphocytes that have a major effector function in local immune responses. While their immunological functions in many inflammatory diseases are well established, comparatively little is still known about their roles in kidney homeostasis and disease. Our understanding of kidney NK cells is rapidly evolving, with murine studies highlighting the functional significance of NK cells in acute and chronic forms of renal disease. Recent progress has been made in translating these murine findings to human kidneys, with indications of NK cell subset-specific roles in disease progression in both native and allograft kidneys. Clearly, a better understanding of the molecular mechanisms driving NK cell activation and importantly, their downstream interactions with intrinsic renal cells and infiltrating immune cells is necessary for the development of targeted therapeutics to halt disease progression. In this review, we discuss the properties and potential functions of kidney NK cells.

## Introduction

Kidney disease is a major public health problem, affecting ~10% of populations in industrialized countries (1). Both acute kidney injury (AKI) and chronic kidney disease (CKD) are increasing worldwide (2). Progression of chronic kidney damage often leads to end stage renal disease with the need for renal replacement therapy (dialysis or transplantation), resulting in significant morbidity and mortality for affected patients.

Regardless of the diverse etiologies underlying AKI and CKD, the immune system is an important determinant in the initiation of most forms of kidney injury. Moreover, chronic inflammation in the kidney is a major driver of CKD progression, not only in autoimmune kidney disease and allograft rejection, but also in metabolic and ischemic renal injury (3, 4). A multitude of studies have provided convincing evidence that conventional T lymphocytes, reactive to classical major histocompatibility complex (MHC)-peptide antigen complexes, are important drivers of immune-mediated kidney pathology (5). In recent years, however, the role of innate and innate-like lymphocyte subsets in the progression of renal disease is beginning to be unraveled (4).

NK cells are one of the specialized subpopulation of innate lymphocytes that, in addition to being critical in anti-viral and tumor defense (6) play significant roles in regulating homeostasis and inflammatory processes in peripheral tissues (7). Although a number of reports point to an important role for NK cells in renal injury, our understanding of NK cell immuno-biology in kidney health and disease is still very limited. Here, we review the current knowledge of NK cells in different forms of acute and chronic kidney injury and, wherever possible, relate the functional evidence provided from studies of experimental animal models of renal disease to observations made in humans.

## NK Cell Subsets

Human NK cells are classically defined as CD3^−^/CD56^+^/CD335 (NKp46)^+^ mononuclear cells that can be subcategorized based on expression levels of CD56 (neural cell adhesion molecule, NCAM) into low density (CD56^dim^) and high density (CD56^bright^) subsets (8). The two subsets are discriminated by their distribution, phenotype and function. CD56^dim^ NK cells are the dominant subset in peripheral blood (9). They express high levels of CD16 (FcγRIII), can express CD57 (a marker of terminal differentiation) and behave as potent cytotoxic effector cells (10–12). In contrast, CD56^bright^ NK cells are preferentially enriched in secondary lymphoid and peripheral tissues (7, 13). CD56^bright^ NK cells are CD16^−/low^ and mediate immune responses by secreting proinflammatory cytokines [e.g., interferon (IFN)-γ and tumor necrosis factor (TNF)-α] (10, 14).

Although murine NK cells show some similarities to the human system with regard to development, maturation, and activation, there are important differences that impact translation of functional studies of NK cell biology from mice to men (6). NK cells in mice are commonly defined by expression of NK cell receptors, such as NKp46 and/or CD161 (NK1.1), and presence of the transcription factors Eomesodermin (Eomes) and T-box expressed in T cells (T-bet). During maturation, they upregulate expression of CD11b and downregulate CD27, while the expression of distinct sets of inhibitory and activating Ly49 receptors marks functional licensing of mature NK cells (6). Similar to humans, distinct tissue-resident and recirculating, “conventional” NK cell subsets have been demonstrated in murine non-lymphoid organs, such as the liver and kidney, differing in transcriptional profile and functional capacity (15, 16). It is noteworthy that tissue-resident NK cell populations share important characteristics with group 1 innate lymphoid cells (ILC1s) that also express NK cell receptors, as well as the transcription factor T-bet, and produce IFN-γ upon stimulation. However, lack of Eomes expression and cytolytic activity separates ILC1s from closely related NK cell populations (17).

## Regulation of NK Cell Responses

NK cells are tightly controlled by a spectrum of both inhibitory and activating receptor:ligand interactions (as recently reviewed in the literature (6, 18). Under normal conditions, healthy cells express MHC class I molecules that engage the inhibitory receptors expressed on NK cells, delivering a negative signal and inhibiting NK cell activity. However, in diseased conditions, damaged and virally infected cells display reduced or aberrant MHC class I and/or express cellular stress ligands that engage with activating receptors on NK cells. In turn, these activation signals lead to NK cell production of inflammatory cytokines and cytotoxic activity (19). Another important pathway for NK cell activation is mediated by cytokines, predominantly derived from myeloid cells, e.g., dendritic cells and macrophages, during inflammatory responses. In particular, IL-12, acting in synergy with other cytokines, such as IL-18, IL-15, and IL-2, can stimulate NK cells to produce IFN-γ and other pro-inflammatory mediators and enhance their cytolytic activity (6).

## Kidney NK Cells Under Steady-State Conditions

Most of our knowledge regarding human NK cells is derived from studies in peripheral blood. However, it is now established that NK cells populate most healthy lymphoid and non-lymphoid organs of the human body, including the kidney. In fact, NK cells constitute a large fraction of total lymphocytes in healthy human kidneys (~25% of lymphocytes) (13). Both CD56^bright^ and CD56^dim^ NK cell subsets have been identified within the NK cell compartment of healthy human kidneys (13, 20). In particular, a proportional enrichment of CD56^bright^ NK cells is observed within healthy human kidneys (~37% of total NK cells) as compared with peripheral blood (<10% of total NK cells) (13).

It still remains unclear whether these human NK cells represent a tissue-resident lymphocyte population permanently retained *in situ* or a circulating lymphocyte population that is transiently recruited to the kidney. In humans, the expression of CD69 (a C-lectin receptor) has been used to discriminate tissue-resident from circulating lymphocytes (21–23). Our group recently reported the expression of CD69 on human NK cells (predominantly on CD56^bright^ NK cells) in healthy kidney tissue (20). Based on this initial indication of tissue residency, we speculate that human NK cells in healthy kidneys serve as sentinels to maintain barrier integrity and protect against pathogens, as has been suggested for tissue-resident NK cells in other human peripheral organs (7, 24–26).

The concept of a specialized NK cell subset that resides in the kidney tissue and is characterized by minimal exchange with its recirculating counterparts is supported by a recent study in mice. Using a parabiosis approach, a technique in which the blood circulations of two animals are surgically anastomosed, investigators showed that the murine kidney harbors two distinct populations of NK cells: tissue-resident (tr) NK cells with the surface marker combination CD49a^+^CD49b^−^, representing ~20% of the total NK cell pool in the kidney, and conventional (c) NK cells which are CD49a^−^CD49b^+^ (16). The kidney-residing trNK cells displayed a surface marker profile distinct from cNK cells, did not require the cNK cell transcription factor NFIL3 for their development, partially depended on T-bet expression and, most importantly, were of functional relevance in a mouse model of ischemic AKI (see below) (16). However, whether these trNK cells play a role in maintaining kidney homeostasis in the steady-state or serve as a first line of defense against invading pathogens remains to be elucidated.

## NK Cells in Ischemic AKI

AKI is a clinical condition defined by acute impairment of kidney function, caused by heterogeneous etiologies including ischemia, sepsis and toxic insults. The most common morphology of (severe) AKI is acute tubular necrosis (ATN). Immunohistological examinations of NK cells in human ATN are limited because clinical practice is not to biopsy when the impairment is expected to be time limited (27). Despite this, there is evidence that NK cells do indeed participate in AKI due to ATN in humans. Highlighting their potential pathogenic function, NK cells have been shown to directly kill human tubular epithelial cells (TECs) exposed to hypoxic conditions mimicking ischemic AKI *in vitro* (28). This cytotoxic function was dependent on the direct interaction of activating NKG2D receptor on NK cells and its ligand MICA expressed on TECs.

**Figure 1:**
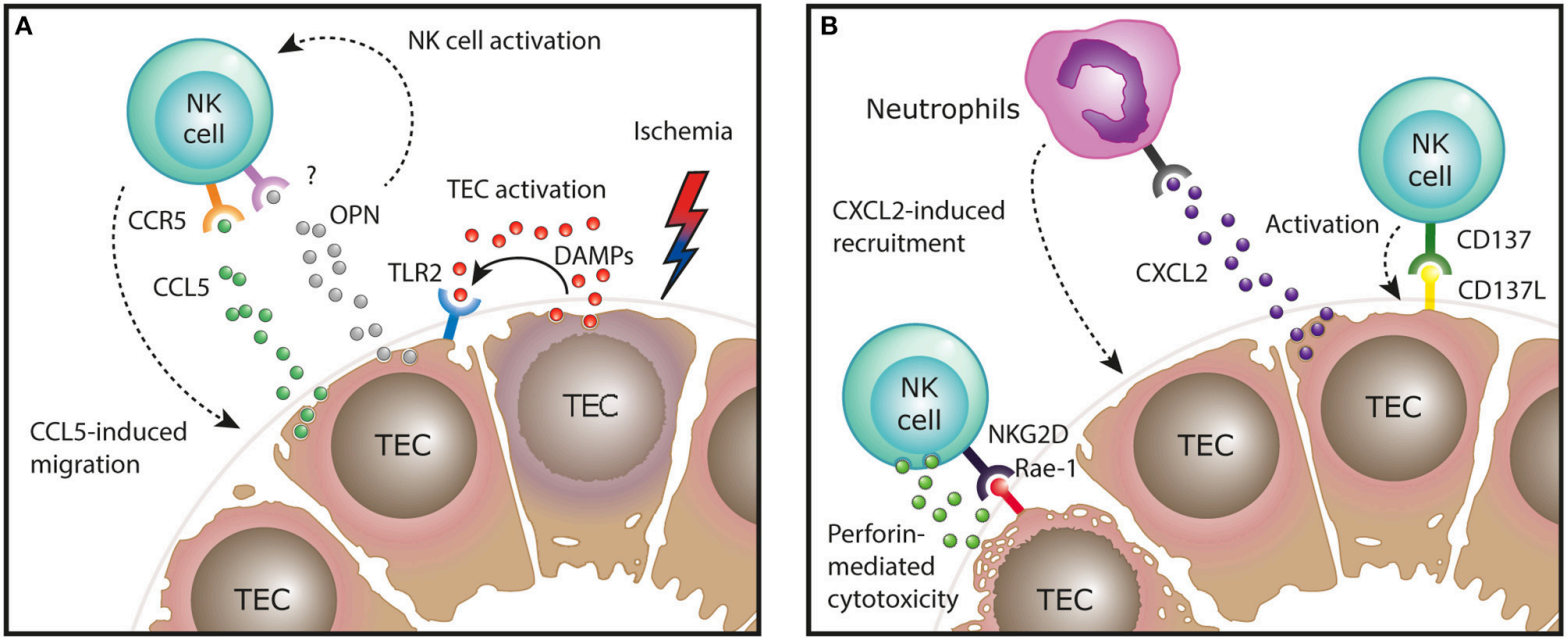
Function of NK cells in the ischemia/reperfusion mouse model of AKI. **(A)** After ischemic injury, tubular epithelial cells (TECs) release endogenous damage-associated molecular pattern (DAMPs) that activate surrounding TECs via TLR2 to express CCR5 ligands, mediating NK cell recruitment. In addition, production of osteopontin (OPN) by injured TECs activates NK cells and indirectly regulates their recruitment, by a yet unknown mechanism. **(B)** After recruitment to the areas of ischemic injury, NK cells can engage in direct interaction with activating molecules expressed on the damaged epithelium. Activation of NK cells by these ligand: receptor interactions, such as NKG2D on NK cells and Rae-1 on TECs, results in perforin-dependent TEC killing. Interaction of CD137L on TECs with CD137^+^ NK cells results in the induction of CXCL2 expression in TECs, leading to neutrophil recruitment and immune-mediated progression of tubular damage.

In mice, the kidney ischemia/reperfusion model has been used in several studies to investigate the role of NK cells in the induction and regeneration of ischemic ATN *in vivo*. In this model, CD49b^+^NKG2D^+^ cNK cells were shown to promote tubular injury induced by the ischemic insult, a finding that depended on NK cell expression of the cytotoxic effector molecule perforin, but was independent of IFN-γ production by NK cells (29). Mechanistically, in line with the human data, activated NK cells were able to directly kill TECs expressing the activating NKG2D ligand Rae-1 *in vitro* (29). It was further shown that ischemic injury of TECs *in vivo* upregulates their expression of Rae-1 and other stress molecules, such as the costimulatory molecule CD137L (30). Interaction of CD137L on TECs with CD137^+^ NK cells resulted in the induction of CXCL2 expression in TECs, leading to neutrophil recruitment and immune-mediated progression of tubular damage (Figure 1) (30).

TECs are also instrumental in the initial recruitment of NK cells to the kidney in ischemic injury. By expressing molecules that induce NK cell chemotaxis, such as CCR5 ligands (e.g., CCL5) and osteopontin, TECs direct NK cells toward areas within the kidney tissue where they can engage in direct interaction with the damaged epithelium (31, 32). The production of CCR5 ligands by TECs was induced by TLR2 signaling, indicating that endogenous TLR2 ligands (damage-associated molecular patterns, DAMPs) released during cell death are sufficient to trigger this pro-inflammatory cascade (Figure 1) (31).

The question of which specific NK cell subset (trNK cells vs. cNK cells) in the mouse kidney possesses pathogenic potential in ischemic AKI was addressed by another recent study harnessing the differential expression of glycolipid asialo GM1 (AsGM1)—being highly expressed on cNK cells, but low on kidney trNK cells—to dissect the functional role of the two subsets (16). Accordingly, anti-AsGM1 antibody depletion preferentially targeted cNK cells, but spared a significant number of trNK cells in the kidney. By comparing total NK cell depletion (trNK cells and cNK cells by using an anti-NK1.1 antibody) to “selective” cNK cell depletion (using an anti-AsGM1 antibody), the authors concluded that predominantly trNK cells mediate tubular damage in ischemic AKI. Taken together, these findings demonstrate that after TEC-mediated recruitment of NK cells to the kidney, specific ligand-receptor interactions between damaged TECs and NK cells not only lead to direct perforin-mediated TEC killing, but initiate an innate inflammatory cascade that promotes immune-mediated injury in ischemic AKI (Figure 1).

## NK Cells in Glomerulonephritis and Other Forms of CKD

**Figure 2:**
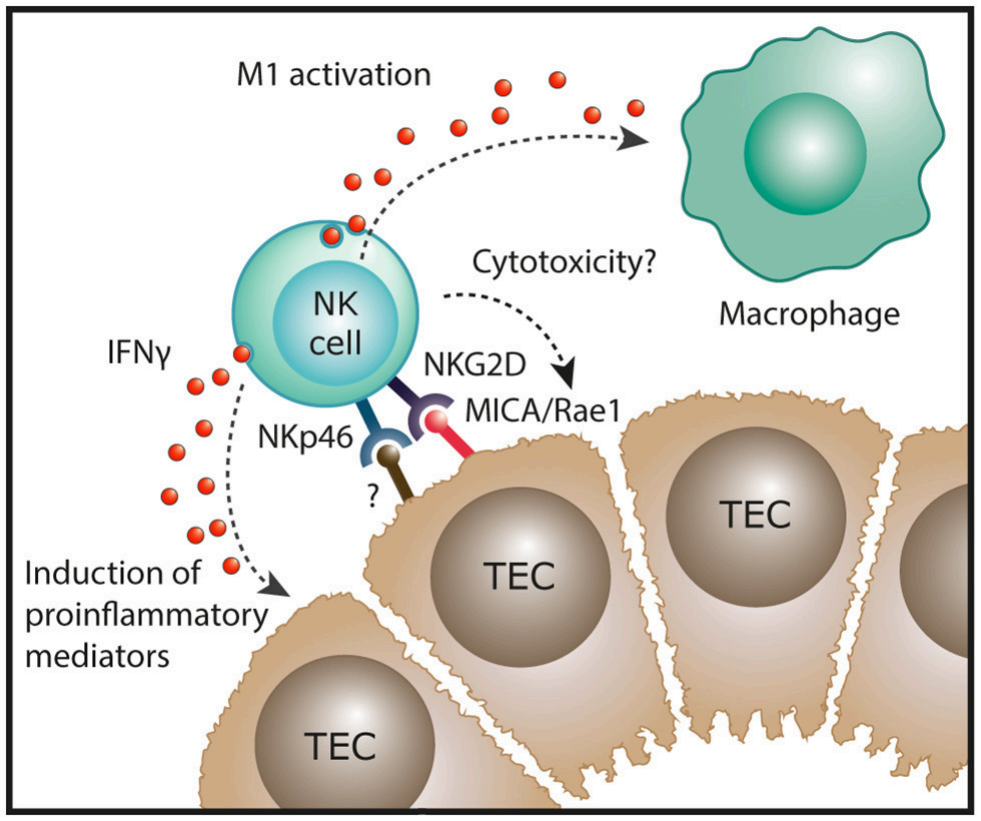
Potential function of NK cells in chronic kidney disease. In human kidney fibrosis, NK cells reside in the tubulointerstitium and express the NK cell receptor NKp46 that can recognize stressed cells. In addition, the NKG2D ligand MICA is upregulated in tubular epithelial cell (TECs) of patients with lupus nephritis. In settings of chronic inflammation, NK cells could exert direct cytotoxic effects on damaged tubular epithelial cells. Moreover, kidney NK cells have been shown to produce IFN-γ in human CKD which could induce proinflammatory mediators in renal parenchymal cells and promote “classical” M1 activation of macrophages, both resulting in progression of renal inflammation.

CKD describes the progressive loss in renal function over a period of at least 3 months and is characterized, irrespective of its origins, by inflammatory injury and fibrosis within the tubulointerstitial compartment (33). Initial immunohistochemical (IHC)-based investigations reported the presence of interstitial NK cells (CD56^+^, CD57^+^ or CD16^+^ cells) in native kidney biopsies from patients with IgA nephropathy (34) and crescentic glomerulonephritis (35). However, these early IHC studies were methodologically limited to single-antigen labeling assays. Indeed, single staining for these antigens lacks the specificity to identify human NK cells, given the broader expression of these individual markers on T cells in kidney tissue. For instance, Uchida et al. have recently suggested that human CD56^+^ T cells are integral to the processes that mediate kidney injury (36). In addition, single-antigen IHC is insufficient to directly identify human NK cell subsets that can only be unequivocally identified by labeling multiple cell surface antigens. Therefore, our group has recently extended these earlier investigations by using a multi-parameter, flow-cytometric-based approach to evaluate NK cell subsets in a cohort of patients with different forms of CKD. In this study, Law et al. demonstrated significant correlations between tubulointerstitial NK cell numbers, in particular CD56^bright^ NK cells, and the histological severity of interstitial fibrosis (20). This suggests that NK cells play a unique role in progression to CKD, regardless of the underlying etiology of kidney disease. Indeed, our group also examined the functional capabilities of these kidney NK cells, identifying CD56^bright^ NK cells as an important source of proinflammatory cytokine IFN-γ in the fibrotic kidney (20). Recent evidence of strong expression of the activating NKG2D ligand MICA in TECs of patients with chronic lupus nephritis (37) provides a possible mechanistic pathway of kidney CD56^bright^ NK cell activation in immune-mediated kidney disease (Figure 2).

Similar to the initial human studies, early investigations in rodent models were reliant on immunohistochemistry to discriminate NK cells in kidney leukocyte infiltrates in immune-mediated glomerular disease. While some studies suggested that NK cells were an important leukocyte subset infiltrating the kidney in glomerulonephritis models (38, 39), others could not confirm this finding (40–42). However, these discrepancies might easily be explained by technical limitations in detection of NK cells and variations in the models used. Another study reported an upregulation of the murine NKG2D ligand Rae-1 and its receptor, as well as increased numbers of CD49b^+^ NK cells, in progressive glomerulosclerosis induced by injection of Adriamycin into BALB/c mice, a widely used mouse model for proteinuric CKD. However, in a functional approach, anti-AsGM1 antibody-mediated depletion of cNK cells or impaired function of NK cells in NOD-SCID mice did not alter disease severity in this model (43). Since we are now aware that the anti-AsGM1 antibody only effectively depletes cNK cells in the kidney, while sparing most of the trNK cells (16) it still remains unclear whether the trNK subset might play a role in chronic glomerular scarring.

The involvement of NK cells in autoimmune glomerular disease was suggested by a more recent study investigating NK cell phenotype and activity in the MRL-*lpr* mouse model of lupus nephritis. Upregulation of NKG2D ligands was evident specifically in the glomeruli of mice with lupus nephritis and correlated with glomerular accumulation of NKp46^+^ cells (37). The NK cells infiltrating the kidney in diseased MRL-*lpr* mice showed a mature/activated phenotype and were able to produce IFN-γ in response to IL-12/IL-15 stimulation, pointing to a potential proinflammatory role of NK cells in murine lupus nephritis.

In conclusion, while NK cells are likely to play a role in interstitial renal fibrosis in CKD (in humans), the evidence to support a substantial role of NK cells in glomerulonephritis is still scarce and detailed functional analyses of kidney NK cell subsets in glomerulonephritis with state-of-the-art methods are still missing.

## NK Cells in Kidney Allograft Rejection

Kidney transplantation is the preferred treatment option for end-stage renal disease (ESRD) patients. However, despite improvements in immunosuppressive regimens, transplant rejection continues to contribute to loss of graft function and eventual graft loss (44). Kidney allograft rejection can be classified pathologically into two types: T cell-mediated rejection (TCMR) and antibody-mediated rejection (ABMR) (45). TCMR is a tubulointerstitial process mediated by host alloreactive lymphocytes targeting donor human leukocyte antigen (HLA) molecules, whilst ABMR is a process of microvascular inflammation (glomerulitis, peritubular capillaritis) driven by donor-specific antibodies (DSA) acting on the allograft endothelium (46–48). Human NK cells have attracted interest as key immunological players in both TCMR and ABMR.

Gene expression profiling of human kidney allografts identified high levels of NK cell transcripts in TCMR biopsies, suggesting a distinct role for NK cells in this tubulointerstitial disease process (49). These transcriptomic data have been validated by IHC-based investigations reporting significant associations between interstitial NK cells and acute TCMR (50, 51). However, these IHC-based studies have identified NK cells based solely on the expression of single antigens (CD56, CD57, or CD16) that cannot unequivocally define this innate lymphocyte population or allow the evaluation of discrete NK cell subsets. Future investigations incorporating multi-color flow cytometric approaches are essential to specifically identify and examine the function of NK cell subsets in human TCMR. A potential role for human NK cells in TCMR pathology may be to secrete proinflammatory cytokines (e.g., IFN-γ, TNF-α) that, in turn, are capable of: (1) inducing chemokines which recruit alloreactive T cells (52) and (2) up-regulating HLA alloantigens on target donor cells to make them more susceptible to cytotoxic killing (53).

**Figure 3:**
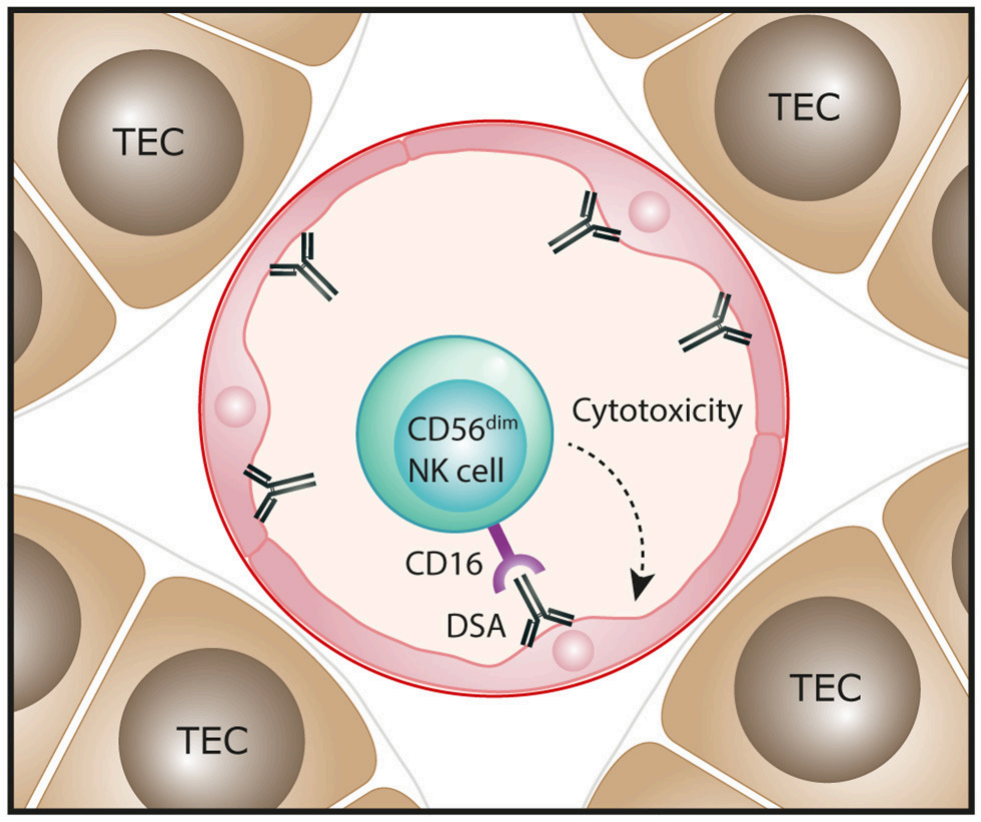
Proposed function of NK cells in human antibody-mediated renal allograft rejection. In antibody-mediated rejection, donor-specific antibodies (DSA) bind to allograft endothelial cells. The interaction of these antibodies with the Fc receptor CD16 that is highly expressed on the CD56^dim^ NK cell subset could trigger antibody-dependent cell-mediated cytotoxicity directed against endothelial cells, resulting in microvascular injury of the kidney allograft.

Molecular assessments of ABMR in human kidney transplants have also been performed, with transcripts representing NK cells shown to be highly associated with ABMR pathology and the presence of DSA (49, 54–57). Single marker IHC-based evaluations have reported NK cells (identified as CD56^+^ or NKp46^+^ cells) in the peritubular capillaries of ABMR biopsies, consistent with an effector role for these innate cells in ABMR microvascular injury (50, 56, 57). In particular, Shin et al. observed that numbers of NK cells were significantly associated with chronic active ABMR, but not with acute ABMR (50). Emerging transcriptomic evidence indicates that NK cell activation in ABMR biopsies is specifically mediated via IgG Fc receptor CD16 triggering (58). Collectively, these findings have led to a proposed pathogenic function for NK cells in human ABMR, whereby DSA bound to allograft endothelial cells will engage with CD16 on NK cells to induce a mechanism of antibody-dependent cell-mediated cytotoxicity directed against the allograft (Figure 3). This model specifically implicates the CD16-expressing CD56^dim^ NK cell subset in driving ABMR pathology and will be an area for future clinical investigation.

The experimental evidence from mouse studies also assigns an important role for NK cells in chronic renal allograft rejection. In a C57BL/6 parent to C57BL/6 x BALB/c F1 kidney transplant model, in which acute rejection is prevented by T cell tolerance to donor MHC, while chronic graft rejection still occurs, CD49b^+^ cNK cells were shown to infiltrate the renal allograft. Intriguingly, chronic allograft injury in this model was still present in *Rag1*^−/−^ mice, a model completely lacking T and B cells, but significantly ameliorated in *Rag1*^−/−^ mice that were also depleted of cNK cells by injection of anti-AsGM1 antibody (59). Another recent study addressed the role of NK cells in a unique mouse model of ABMR, in which transplantation of MHC-mismatched kidney allografts into *Ccr5*^−/−^ mice results in the development of DSA and signs of ABMR within 2–5 weeks (60). In this model, early depletion of the total NK cell pool by anti-NK1.1 therapy (in the absence of CD8^+^ cytotoxic T cells in *Cd8*^−/−^*Ccr5*^−/−^ mice) resulted in reduction of IFN-γ, perforin, granzyme B, and other proinflammatory molecules in the renal transplants. Most importantly, however, with sustained depletion of NK cells in *Cd8*^−/−^*Ccr5*^−/−^ mice, long-term survival of about 40% of renal transplants could be achieved (61).

## Concluding Remarks

The collective findings from experimental mouse models and cellular/molecular studies of human kidney specimens highlight the complex functions of kidney NK cells during homeostatic and pathological conditions. Indeed, we have presented evidence that NK cells in the kidney are a heterogeneous population of innate lymphocytes with subset-specific functional roles. Further evaluation of the kidney NK cell compartment in native and allograft models will enable the development of therapeutic approaches that specifically target the recruitment or triggering of discrete NK cell subsets dependent on the pathological conditions.

## Author Contributions

Each author has participated sufficiently in the work to take public responsibility for the content. J-ET, CR, HH, and AK drafted, revised and approved the final version of the manuscript.

### Conflict of Interest Statement

The authors declare that the research was conducted in the absence of any commercial or financial relationships that could be construed as a potential conflict of interest.
